# *Lactiplantibacillus* sp. LP03 alleviates pulmonary fibrosis by modulating gut microbiota and elevating host palmitoylethanolamide to suppress TGF-β1/Smad2/3-mediated EMT

**DOI:** 10.3389/fmicb.2025.1659142

**Published:** 2025-09-09

**Authors:** Yuhao Zhou, Meina Duan, Xiaoye Fan, Yuzhe Zhao, Chao Wang, Tingting Wang, Shucheng Hua

**Affiliations:** ^1^Department of Respiratory Medicine, Center for Pathogen Biology and Infectious Diseases, Jilin Provincial Key Laboratory for Individualized Diagnosis and Treatment of Pulmonary Diseases, The First Hospital of Jilin University, Changchun, China; ^2^State Key Laboratory for Diagnosis and Treatment of Severe Zoonotic Infectious Diseases, The First Hospital of Jilin University, Changchun, China; ^3^Department of Traditional Chinese Medicine (TCM), Zibo First Hospital, Zibo, China; ^4^Department of Infectious Diseases, Zibo First Hospital, Zibo, China

**Keywords:** *Lactiplantibacillus*, gut microbiota, palmitoylethanolamide, TGF-β1/Smad2/3 signaling pathway, EMT, idiopathic pulmonary fibrosis

## Abstract

**Introduction:**

Idiopathic pulmonary fibrosis (IPF) is a progressive and fatal interstitial lung disease with limited treatment options. Emerging evidence suggests that gut microbiota dysbiosis contributes to pulmonary disorders, underscoring the therapeutic potential of probiotics.

**Methods:**

Three Lactobacillus strains—Lactiplantibacillus sp. LP03 (LP03), Levilactobacillus brevis LB06, and Loigolactobacillus coryniformis LC0—were isolated from Chinese sauerkraut juice and evaluated in a bleomycin (BLM)-induced mouse model of pulmonary fibrosis. Gut microbiota composition was analyzed, and serum metabolomics profiling was performed to explore underlying mechanisms. Further, the therapeutic role of palmitoylethanolamide (PEA) was assessed both *in vivo* and *in vitro*.

**Results:**

Among the three strains, LP03 exhibited the most pronounced antifibrotic effects, including reduced mortality, systemic inflammation, lung coefficient, interstitial thickening, and collagen deposition, as well as inhibition of BLM-induced epithelial-to-mesenchymal transition (EMT). LP03 treatment restored gut microbial balance, notably increasing beneficial genera such as Ligilactobacillus and Akkermansia. Metabolomic analysis revealed enhanced lipid metabolism, especially in glycerophospholipid and fatty acid pathways, and elevated serum PEA levels. Oral PEA supplementation independently alleviated fibrosis, while mechanistic studies demonstrated that PEA mitigated fibrosis by inhibiting EMT through suppression of the TGF-β1/Smad2/3 signaling pathway.

**Discussion:**

These findings highlight LP03 as a promising probiotic candidate for pulmonary fibrosis therapy. Its therapeutic effects are mediated by remodeling of the gut microbiota and elevation of systemic PEA, which in turn regulates fibrotic signaling pathways.

## 1 Introduction

Idiopathic pulmonary fibrosis (IPF) is a rare, progressive, and etiologically uncertain pulmonary disorder characterized by the scarring and fibrosis of lung tissue, leading to dyspnea and impaired oxygenation (Maddali et al., 2024; Lu et al., 2025). Despite advances in understanding its pathogenesis, IPF remains a clinical challenge with limited therapeutic options and a poor prognosis (Gonnelli et al., 2024; Gupta et al., 2024).

Epithelial-mesenchymal transition (EMT) is a dynamic biological process wherein epithelial cells acquire mesenchymal fibroblast-like characteristics, including diminished intercellular adhesion and enhanced migratory capacity (Marconi et al., 2021). In lung tissue biopsies from IPF patients, the presence of epithelial cells exhibiting mesenchymal characteristics provides direct histological evidence of EMT (Kim et al., 2006). Accumulating evidence demonstrates EMT’s critical involvement in pulmonary fibrosis pathogenesis and its potential as a therapeutic target (Ding et al., 2023; Ni et al., 2024; Zhou S. et al., 2024). Emerging studies further indicate that systemic inflammation and immune dysregulation significantly contribute to disease progression (Mutsaers et al., 2023; Wang et al., 2023; Chen T. et al., 2024).

Moreover, the role of the gut-lung axis, a bidirectional communication network between the gastrointestinal tract and the lungs, has garnered increasing attention in the context of inflammatory and fibrotic lung diseases (Sun et al., 2024; Zhou et al., 2025). The gut microbiota is a dynamic ecosystem that influences host metabolism, immune function, and inflammatory responses (Brown et al., 2025; Zhang et al., 2025). Disruptions in gut homeostasis, commonly referred to as dysbiosis, have been implicated in a range of pulmonary conditions, including asthma, chronic obstructive pulmonary disease (COPD), and pulmonary fibrosis (Gong et al., 2021; Qu et al., 2022; Guo et al., 2024; Ruan et al., 2024; Wang et al., 2024).

Probiotics are defined as “live microorganisms that, when administered in adequate amounts, confer a health benefit on the host” (Hill et al., 2014). As one of the most extensively studied probiotic genera, *Lactobacillus* has been reported to exert antifibrotic effects through diverse mechanisms across different disease models—for example, modulation of immune cell responses in liver fibrosis and chronic kidney disease–associated renal fibrosis, enhancement of butyrate production in peritoneal fibrosis, and regulation of long non-coding RNA expression in radiation-induced pulmonary fibrosis (Kim et al., 2022; Ju et al., 2023; Wu et al., 2023; Yang et al., 2023). However, to the best of our knowledge, no studies have directly investigated the role of *Lactobacillus* in bleomycin (BLM)-induced pulmonary fibrosis. This gap in the literature underscores the novelty of the present work and provides a strong rationale for evaluating whether *Lactobacillus* can similarly mitigate fibrotic progression in the lung.

Sauerkraut, a traditional fermented vegetable product, serves as a rich source of diverse potential probiotic microorganisms (Xu et al., 2020; Chen Q. et al., 2024; Yang et al., 2024). From traditional fermented sauerkraut juice in Northeast China, we isolated and characterized three candidate probiotic strains that satisfied phenotypic criteria for gastrointestinal survival (see section “2 Materials and methods”). This work advances our understanding of gut-lung axis modulation in fibrosis and positions *Lactiplantibacillus* sp. LP03 (LP03) as a novel microbial therapeutic candidate for IPF. We hypothesize that LP03 attenuates BLM-induced pulmonary fibrosis through modulation of gut microbiota and elevation of palmitoylethanolamide (PEA), thereby suppressing the TGF-β1/Smad2/3-mediated EMT pathway. Using a BLM-induced pulmonary fibrosis murine model, we systematically evaluated the therapeutic potential and mechanistic basis of LP03.

## 2 Materials and methods

### 2.1 Chemicals and reagents

Bleomycin sulfate was purchased from Selleck (Shanghai, China). Primary antibodies against GAPDH, fibronectin, collagen I, α-SMA, E-cadherin, N-cadherin, vimentin, Smad-2, Phospho-Smad2 (p-Smad2), Smad-3, and Phospho-Smad3 (p-Smad3) were sourced from ABclonal (Wuhan, China). The BCA protein assay kit was obtained from Beyotime Bio (Shanghai, China). High-Sig ECL western blotting substrate was purchased from Tanon (Shanghai, China). The MTT Cell Proliferation and Cytotoxicity Assay Kit was acquired from Solarbio (Beijing, China). ELISA kits for IL-6, IL-1β, and TNF-α were obtained from Boster (Wuhan, China). The Superoxide Dismutase (SOD) Activity Assay Kit, Malondialdehyde (MDA) Content Assay Kit, and Reduced Glutathione (GSH) Content Assay Kit were provided by Nanjing Jiancheng Bioengineering Institute (Nanjing, China). Ultra-micronized PEA was obtained from Epitech Group SpA (Saccolongo, Padua, Italy). Recombinant Human TGFβ1 protein was purchased from R&D Systems (Minnesota, United States). The TGF-β/Smad pathway activator, SRI-011381, was sourced from MedChemExpress (New Jersey, United States). All cell culture reagents were obtained from Gibco (Grand Island, NY, United States) and Solarbio (Beijing, China). Trizol reagent was sourced from Takara (Dalian, China). The PrimeScript RT Reagent Kit and the SYBR Premix Ex Taq Kit were purchased from Yeasen (Shanghai, China).

### 2.2 Isolation, identification, and acid/bile salt tolerance evaluation of *Lactobacillus* strains

The raw sauerkraut juice was serially diluted in sterile physiological saline and plated onto de Man, Rogosa, and Sharpe (MRS) agar using the streak plate method. Following incubation until distinct white colonies appeared, individual colonies were randomly selected and subjected to two successive rounds of purification. The bacterial isolates were taxonomically classified through 16S ribosomal DNA (rDNA) sequencing with subsequent EzBioCloud database alignment (Yoon et al., 2017; Chalita et al., 2024), identifying three distinct strains: *Lactiplantibacillus* sp. LP03 (LP03), *Levilactobacillus brevis* LB06 (LB06), and *Loigolactobacillus coryniformis* LC02 (LC02). Following confirmation of LP03’s anti-fibrotic efficacy against BLM-induced pulmonary fibrosis in mice, the strain was deposited in the China Center for Type Culture Collection (CCTCC) with the official designation *Lactiplantibacillus* sp. ZYHL3 (CCTCC No. M 2025127).

To evaluate gastrointestinal survival capacity—a critical characteristic of probiotics—we assessed acid tolerance (pH 2.0–5.0 for 4 h, simulating typical gastric retention time) and bile salt resistance (0.03%–0.3% bile salt for 4 h, representing small intestinal transit duration). These experiments followed optimized protocols, incorporating modifications to pH ranges, bile salt concentrations, and incubation times to more closely simulate physiological conditions (Zhao et al., 2023, 2025; Xiong et al., 2024). All three *Lactobacillus* strains maintained > 50% viability under stringent conditions (pH 2.0 and 0.3% bile salt) in triplicate experiments (see Supplementary Figure 1).

### 2.3 Animal treatment

All animal procedures were conducted in strict accordance with the ethical standards approved by the Animal Ethics Committee of the First Hospital of Jilin University (Approval No. JDYY20240346). Experimental protocols were authorized by the relevant licensing authority and complied fully with institutional regulations and the ARRIVE guidelines.

A total of 8 weeks-old male C57BL/6J mice (20–22 g) were purchased from Jilin Qianhe Technology Industrial Co., Ltd. (Changchun, Jilin, China) and housed under specific pathogen-free conditions at the animal research facility of the First Hospital of Jilin University (Changchun, Jilin, China). The mice were acclimatized for 1 week before beginning the experiments and were given *ad libitum* access to food and water (Zhu et al., 2021a,b). Following acclimatization, mice were randomly allocated into five groups (*n* = 13 per group): (1) Control (Con) group: normal saline treatment; (2) Model group (BLM group): BLM-induced pulmonary fibrosis; (3) LP03 group: BLM + LP03; (4) LB06 group: BLM + LB06; and (5) LC02 group: BLM + LC02. To establish a pulmonary fibrosis model, mice were administered 5 mg/kg of BLM via endotracheal injection under anesthesia with tribromoethanol, following established protocols (Peng et al., 2020; Ding et al., 2022; Li G. et al., 2023; Zeng et al., 2024). Mice in the Con group received an intratracheal instillation of an equal volume of saline solution. The dosage and frequency of our *Lactobacillus* intervention were adapted from existing studies on probiotics in disease treatment and appropriately modified based on the specific context of our research (Duan et al., 2023; Shen et al., 2024; Vasconcelos et al., 2025). Seven days prior to BLM administration, 10^9^ CFU of LP03, LB06, or LC02, each suspended in 200 μL of saline, were administered by oral gavage once daily, 5 days a week, continuing until day 21 post-BLM administration. Mice in the Con and BLM groups were gavaged with an equal volume of saline at the same time. On day 21, the mice were sacrificed, and lung tissues, bronchoalveolar lavage fluid (BALF), blood, and colonic feces were collected for further analysis.

To further investigate the effects of the differential metabolite PEA on BLM-induced pulmonary fibrosis, mice were assigned to five experimental groups (*n* = 15 per group): (1) Con group; (2) BLM group; (3) PEA (3 mg/kg) group: BLM + PEA (3 mg/kg); (4) PEA (10 mg/kg) group: BLM + PEA (10 mg/kg); and (5) PEA (30 mg/kg) group: BLM + PEA (30 mg/kg). To enhance oral bioavailability, ultra-micronized PEA at various concentrations was dissolved in 1.5% carboxymethyl cellulose in accordance with previous studies (Annunziata et al., 2020; Lama et al., 2021). PEA was administered once daily via oral gavage from day 1 to day 20. On day 21, mice were humanely euthanized via intraperitoneal injection of sodium pentobarbital (50 mg/kg), and samples were subsequently collected for further analysis.

### 2.4 Cell culture and treatment

A549 cells (human type II alveolar epithelial cell line) and MRC5 cells (human embryonic lung fibroblast line) were obtained from the American Type Culture Collection (ATCC, Manassas, VA, United States). Both cell lines were cultured in DMEM medium containing 10% fetal bovine serum (FBS) and 1% penicillin-streptomycin, and maintained at 37 °C in a 5% CO_2_ atmosphere. Upon reaching approximately 80% confluency, cells were digested using 0.25% trypsin/EDTA and seeded into 6-well plates (Song et al., 2016, 2017). The following day, cells were serum-starved in DMEM containing 1% FBS for 12 h and then treated with different compounds (TGFβ1, PEA, SRI-011381).

### 2.5 Hematoxylin and eosin (H&E) staining and Masson’s Trichrome staining

The left lungs of mice were fixed in 4% paraformaldehyde for 24 h, embedded in paraffin, and sectioned into 4 μm thick slices. For H&E staining, sections were deparaffinized using xylene, rehydrated through graded ethanol solutions, and stained with hematoxylin to stain nuclei blue or purple, followed by eosin to stain cytoplasm, collagen, and other structures pink or red. After staining, the slides were dehydrated, cleared, and mounted. For Masson’s Trichrome staining, after deparaffinization and rehydration, the sections were stained with Weigert’s iron hematoxylin to stain nuclei black, followed by Biebrich scarlet acid fuchsin to stain cytoplasm and muscle fibers red. The sections were then differentiated with phosphomolybdic acid, followed by staining with aniline blue to visualize collagen fibers as blue or green. After final dehydration and clearing, the slides were mounted and photographed using light microscopy. All imaging was performed by an assessor blinded to the treatment conditions.

### 2.6 Oxidative stress analysis

The enzymatic activities of superoxide dismutase (SOD) and the levels of malondialdehyde (MDA) and reduced glutathione (GSH) in lung tissue homogenates were measured according to the manufacturer’s protocols.

### 2.7 Cell viability assay

Cell viability was assessed using the MTT Cell Proliferation and Cytotoxicity Assay Kit. A549 and MRC5 cells were seeded into 96-well plates (5 × 10^3^ cells per well) and incubated for 12 h. Cells were treated with increasing concentrations of PEA (0, 0.5, 1, 2, 4, 8, 16, 32, and 64 μM) for 24 h. Following treatment, the medium was removed, and 90 μL of fresh medium was added, followed by 10 μL of MTT solution. After 4 h of incubation at 37 °C, the supernatant was removed, and 110 μL of Formazan solubilization solution was added to each well. The absorbance was measured at 490 nm using a microplate reader.

### 2.8 Western blot analysis

Proteins were extracted from tissue or cell lysates using RIPA buffer containing phosphatase and protease inhibitors. After centrifugation, protein concentrations were quantified using the BCA assay. Equal amounts of protein (25 μg) were separated by SDS-PAGE and transferred to PVDF membranes (Song et al., 2021; Luo et al., 2023). After blocking, the membranes were incubated overnight at 4 °C with primary antibodies, followed by incubation with HRP-conjugated secondary antibodies. Protein levels were visualized using enhanced chemiluminescence reagents, and densitometric analysis was performed using Image J software.

### 2.9 Enzyme-linked immunosorbent assay (ELISA)

Bronchoalveolar lavage was performed with cold saline. The concentrations of IL-6, IL-1β, and TNF-α in the BALF were measured using ELISA kits according to the manufacturer’s protocols.

### 2.10 Immunohistochemistry (IHC)

Lung tissues were initially fixed in 4% paraformaldehyde, followed by a series of dehydration steps prior to paraffin embedding. Sections (4 μm) were deparaffinized, rehydrated, and subjected to antigen retrieval using citrate buffer at 95 °C for 10 min. After antigen retrieval, the sections were incubated in endogenous peroxidase blocking solution at 37 °C for 30 min to inhibit non-specific binding. Subsequently, the sections were immunostained with primary antibodies against vimentin, incubating overnight at 4 °C. Following primary antibody incubation, the sections were treated with biotin-conjugated secondary antibodies at 37 °C for 30 min. Immunoreactivity was visualized using a DAB working solution, and the sections were counterstained with hematoxylin before mounting with a permanent mounting medium. Images of the stained sections were captured using light microscopy, with the imaging process conducted by an assessor blinded to the experimental groups.

### 2.11 Total RNA isolation and reverse transcription quantitative PCR (RT-qPCR)

For the transcriptional analysis of lung tissues or cultured cells, total RNA was extracted using TRIzol reagent, following the manufacturer’s protocol. The RNA was then reverse transcribed into complementary DNA (cDNA) using the PrimeScript RT Reagent Kit. Gene expression levels were quantified via qPCR using the SYBR Premix Ex Taq Kit, with cDNA as the template. GAPDH was employed as an internal control. Relative mRNA expression levels were calculated using the 2^ΔΔ^*^Ct^* method. The primer sequences used in this study are provided in (see Supplementary Table 1).

### 2.12 DNA extraction and 16S rDNA sequencing

Immediately after collection, all fecal samples were frozen at −20 °C and transported on ice to the laboratory. Bacterial DNA was extracted at Novogene Bioinformatics Technology Co., Ltd. using Tiangen kits, following the manufacturer’s protocol. To profile the mouse gut microbiome, the V4 region of the 16S rDNA was amplified and sequenced using universal primers targeting this region, which is conserved across most bacterial species (F: 5′-GTGCCAGCMGCCGCGGTAA-3′; R: 5′-GGACTACHVGGGTWTCTAAT-3′; a unique 6 bp barcode for each sample was incorporated in the reverse primer) (Sonnenburg et al., 2016). Single amplification reactions were performed in 25 μL volumes with 50 ng of template DNA. The PCR conditions were as follows: an initial denaturation at 94 °C for 4 min, followed by 30 cycles of 94 °C for 30 s, 54 °C for 30 s, and 72 °C for 30 s, with a final extension at 72 °C for 5 min. The PCR products were pooled in equimolar concentrations and sequenced on the Illumina HiSeq2500 platform. Alpha- and beta-diversities of the microbiota were analyzed using QIIME 1.9.1 software. The Shannon diversity index and the Simpson diversity index were used as measures of α-diversity, while β-diversity was assessed using weighted UniFrac distances and visualized through principal coordinate analysis (PCoA). Linear discriminant analysis (LDA) effect size (LEfSe) was performed to identify taxa with significant differential abundance among experimental groups. Briefly, a non-parametric Kruskal–Wallis test was used to detect significantly different features, followed by LDA to estimate the effect size of each feature. Only taxa with an LDA score (log10) > 2 and *p* < 0.05 were considered significant. All samples were paired-end sequenced on the Illumina HiSeq X Ten platform (insert size 350 bp, read length 150 bp) at Novogene Bioinformatics Technology Co., Ltd.

### 2.13 Untargeted metabolomics analysis

Untargeted metabolite analysis in serum samples was performed using liquid chromatography-mass spectrometry (LC-MS). Chromatographic separation was conducted on a Thermo Scientific UltiMate 3000 HPLC system equipped with an ACQUITY UPLC BEH C18 column (100 mm × 2.1 mm, 1.8 μm, Waters, United Kingdom) for reversed-phase separation. The column was maintained at 35 °C, and the mobile phase was composed of solvent A (water with 0.1% formic acid) and solvent B (acetonitrile with 0.1% formic acid) at a flow rate of 0.4 mL/min. The gradient elution program was as follows: 0–0.5 min, 5% B; 0.5–7 min, 5% to 100% B; 7–8 min, 100% B; 8–8.1 min, 100% to 5% B; 8.1–10 min, 5% B. A 4 μL injection volume was used for each sample. Metabolites were detected using a high-resolution tandem mass spectrometer (Q-Exactive, Thermo Scientific) operating in both positive and negative ion modes. Precursor ion spectra (m/z 70–1,050) were acquired at a resolution of 70,000, with a target automatic gain control (AGC) of 3e6 and a maximum injection time of 100 ms. Data acquisition was conducted in top 3 mode in data-dependent acquisition (DDA), and fragment ion spectra were collected at a resolution of 17,500, with a target AGC of 1e5 and a maximum injection time of 80 ms.

### 2.14 Wound-healing assay

The wound healing assay was performed as described before (Song et al., 2022). Briefly, A549 and MRC5 cells were seeded in 6-well culture plates and allowed to grow to confluence. Once confluent, a wound was created by scratching the center of the well using a 200 μL pipette tip. The cells were then washed three times with PBS to remove detached cells and debris. The cells were subsequently cultured in DMEM containing 1% FBS, with or without the addition of 10 μM PEA or 10 ng/mL SRI-011381, for an additional 24 h. Microscopic imaging was performed to capture the wound area before and after treatment. The wound healing rate was quantified using ImageJ software (National Institutes of Health, Bethesda, MD, United States) by calculating the difference in scratch area at 0 and 24 h. Wound healing rate (%) = 100 × (scratch area at 0−scratch area at 24 h)/scratch area at 0 h. This experiment was performed in triplicate.

### 2.15 Statistical analysis

Data were analyzed using GraphPad Prism 8.0 and expressed as mean ± standard deviation (SD). One-way ANOVA followed by Tukey’s multiple comparison test was used for statistical comparisons. Kaplan-Meier survival analysis was employed to assess survival data. A *p*-value of < 0.05 was considered statistically significant.

## 3 Results

### 3.1 LP03 attenuates BLM-induced pulmonary fibrosis in mice

**FIGURE 1:**
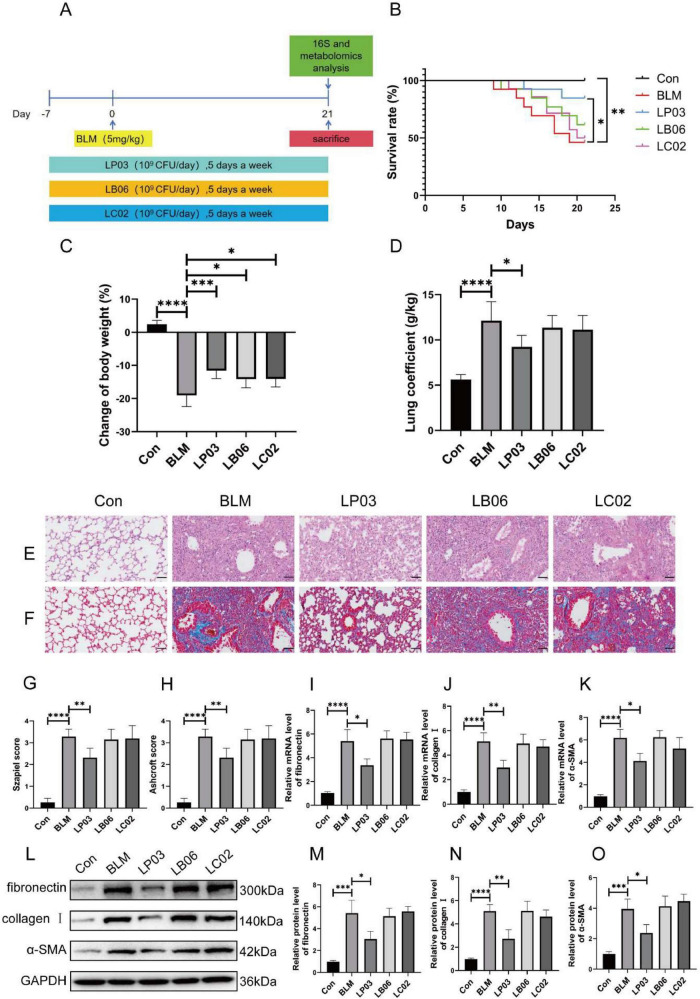
Therapeutic effects of LP03 on bleomycin (BLM)-induced pulmonary fibrosis in mice. **(A)** Experimental design schematic evaluating three *Lactobacillus* strains in pulmonary fibrosis. **(B)** Kaplan-Meier survival curves showing the survival rates of mice in each group throughout the experimental period. **(C)** Endpoint body weight changes across the different groups. **(D)** Lung coefficient of mice at the time of sacrifice. **(E,F)** Representative histopathological images of lung tissues from mice at the study endpoint, stained with H&E and Masson’s trichrome (Scale bar = 50 μm). **(G,H)** Quantitative assessment of lung tissue histology based on Szapiel and Ashcroft scores for H&E and Masson’s trichrome-stained sections, respectively. **(I–K)** RT-qPCR analysis of fibrotic markers (fibronectin, α-SMA, and collagen I). **(L–O)** Western blot quantification of fibronectin, collagen I, and α-SMA. Data are presented as mean ± SD; *p < 0.05, **p < 0.01, ***p < 0.001, ****p < 0.0001 vs. BLM group.

Initially, we explored the effects of these three *Lactobacillus* strains, representing different genera, on BLM-induced pulmonary fibrosis in mice. Figure 1A illustrates the schematic diagram of the *in vivo* study design, investigating the effects of various *Lactobacillus* strains on the BLM-induced pulmonary fibrosis mouse model. Figure 1B shows that LP03 significantly reduced the mortality rate in BLM-treated mice, whereas LB06 and LC02 had negligible effects on survival, highlighting LP03’s distinctive and potential role in improving the prognosis of pulmonary fibrosis. The mice’s body weight was recorded on the first day of the experiment and on the day of euthanasia, showing that all three genera alleviated BLM-induced weight loss, with the LP03 group demonstrating the most significant improvement (Figure 1C). The lung coefficient, defined as the ratio of lung weight to body weight, is a key parameter in animal studies for assessing the extent of pulmonary edema or fibrosis (Zuo et al., 2022; Pan et al., 2024). Compared to the control group, the lung coefficient in the BLM group increased significantly, but subsequently decreased markedly following LP03 administration. In contrast, treatment with LB06 and LC02 had no effect on the lung coefficient (Figure 1D). Importantly, H&E and Masson’s staining of lung tissue revealed that LP03 administration, but not LB06 or LC02, alleviated inflammatory cell infiltration, reduced alveolar wall thickness, and decreased collagen deposition in the lungs of BLM-treated mice (Figures 1E, F). The Szapiel score (Figure 1G) and Ashcroft score (Figure 1H) were used to quantitatively assess the degree of alveolitis and fibrosis, respectively, across the different groups. These findings suggest that LP03 intervention can mitigate the severity of BLM-induced pulmonary fibrosis.

The relative mRNA expression levels of fibronectin, collagen I, and α-SMA—key biomarkers of fibrosis—were assessed by RT-qPCR. The results showed that BLM-induced toxicity significantly upregulated the transcription of all three genes, an effect that was notably reversed by LP03 administration, but not by LB06 or LC02 treatments (Figures 1I–K). Furthermore, western blot analysis revealed that only LP03 treatment significantly attenuated the BLM-induced upregulation of fibronectin, collagen I, and α-SMA at the protein level among the groups (Figure 1L–O). These findings were consistent with the mRNA expression results, further corroborating LP03’s notable anti-fibrotic effect in mice. Collectively, these results suggest that administration of LP03 can mitigate BLM-induced pulmonary fibrosis *in vivo*.

### 3.2 LP03 attenuates BLM-induced EMT process, oxidative stress injury and inflammation in mice

**FIGURE 2:**
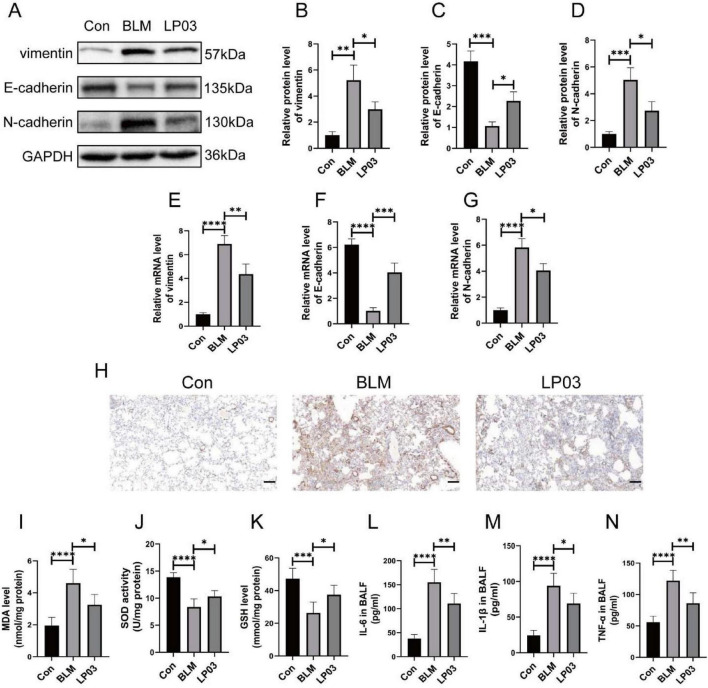
LP03 attenuates epithelial-to-mesenchymal transition (EMT), oxidative stress, and inflammation in a bleomycin (BLM)-induced pulmonary fibrosis mouse model. **(A–D)** Protein levels of vimentin, E-cadherin, and N-cadherin were assessed by western blotting. **(E–G)** mRNA expression levels of vimentin, E-cadherin, and N-cadherin were quantified by reverse transcription quantitative PCR (RT-qPCR). **(H)** Representative immunohistochemical images of vimentin protein expression in mouse lung tissue (Scale bar = 50 μm). **(I–K)** Malondialdehyde (MDA) concentrations, Superoxide Dismutase (SOD) activity, and Glutathione (GSH) levels were measured in homogenized lung tissue. **(L–N)** The levels of inflammatory cytokines IL-6, IL-1β, and TNF-α in bronchoalveolar lavage fluid (BALF) from mice were measured using enzyme-linked immunosorbent assay (ELISA). Data are presented as mean ± SD; *p < 0.05, **p < 0.01, ***p < 0.001, ****p < 0.0001 vs. BLM group.

Accumulating evidence has established EMT as a pivotal mechanism driving pulmonary fibrosis pathogenesis (Kim et al., 2006; Ding et al., 2023; Jin et al., 2024; Ni et al., 2024; Zhou S. et al., 2024). In the present study, we demonstrated that BLM significantly upregulated the protein expression of mesenchymal markers, such as vimentin and N-cadherin, in lung tissue, an effect that was notably reversed by LP03 treatment (Figures 2A, B, D). Conversely, LP03 treatment reversed the BLM-induced reduction in the epithelial marker E-cadherin (Figures 2A, C). A similar trend was observed at the mRNA level, further supporting the Western blot findings (Figures 2E–G). Additionally, the expression of vimentin was further assessed by IHC. As shown in Figure 2H, vimentin levels were elevated in the BLM group, a phenomenon that was reversed following LP03 treatment. Collectively, these results confirm that LP03 effectively mitigates EMT induced by BLM *in vivo*.

Furthermore, we assessed oxidative stress levels by measuring the activity of SOD and the concentrations of MDA and GSH in lung tissue, given the pivotal role of oxidative stress in the pathogenesis of pulmonary fibrosis (Assayag et al., 2021; Liu et al., 2023). SOD is an essential antioxidant enzyme that catalyzes the conversion of superoxide radicals (O2) into hydrogen peroxide (H2O2), thereby protecting cells from oxidative damage. MDA is a product of lipid peroxidation and its levels correlate with cellular oxidative damage. GSH is a tripeptide that acts as a critical intracellular antioxidant, maintaining cellular redox homeostasis. In the present study, the BLM challenge resulted in a significant increase in MDA concentration, whereas LP03 treatment partially restored MDA levels. Conversely, the BLM challenge induced a reduction in pulmonary GSH levels and SOD activity, both of which were notably elevated following LP03 treatment (Figures 2I–K). These findings provide compelling evidence that LP03 exerts a protective effect against oxidative stress in the context of pulmonary fibrosis.

To assess the inflammatory response in BLM-induced pulmonary fibrosis, the expression of inflammatory cytokines in the BALF was measured (Figures 2L–N). Following BLM administration, levels of the inflammatory cytokines TNF-α, IL-6, and IL-1β were significantly elevated. However, after treatment with LP03, the concentrations of TNF-α, IL-6, and IL-1β were notably reduced, highlighting the protective effect of LP03 against the inflammatory response in BLM-induced pulmonary fibrosis.

### 3.3 LP03 attenuates BLM-induced activation of the TGF-β1/Smad2/3 signaling pathway in mice

**FIGURE 3:**
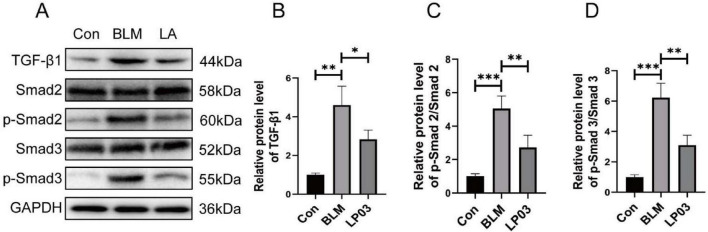
*Lactiplantibacillus* sp. LP03 (LP03) inhibits the TGF-β1/Smad2/3 signaling pathway in lung tissues of a bleomycin (BLM)-induced pulmonary fibrosis mouse model. **(A–D)** Representative western blot images and corresponding quantitative analyses of TGF-β1, phosphorylated Smad2 (p-Smad2), and phosphorylated Smad3 (p-Smad3) protein expression levels. Quantification was performed using ImageJ software. Data are presented as mean ± SD. *p < 0.05, **p < 0.01, ***p < 0.001 vs. BLM group.

The TGF-β/Smad signaling pathway plays a critical role in promoting EMT and is a central pro-fibrotic axis responsible for the aberrant expression and deposition of extracellular matrix (ECM) components in pulmonary fibrosis (Ding et al., 2023; Li G. et al., 2023; Du et al., 2024). To further elucidate the mechanisms underlying the anti-fibrotic effects of LP03, we examined its impact on the TGF-β1/Smad2/3 signaling pathway in a BLM-induced pulmonary fibrosis mouse model. As anticipated, western blot analysis showed significantly elevated levels of TGF-β1 and increased phosphorylation of Smad2 and Smad3 in the BLM group compared to the control group. Notably, treatment with LP03 markedly suppressed the expression of TGF-β1 and the phosphorylation of Smad2/3, indicating effective inhibition of the TGF-β1/Smad signaling cascade (Figure 3).

### 3.4 LP03 partially restores the gut microbiota composition in mice following BLM-induced dysbiosis

**FIGURE 4:**
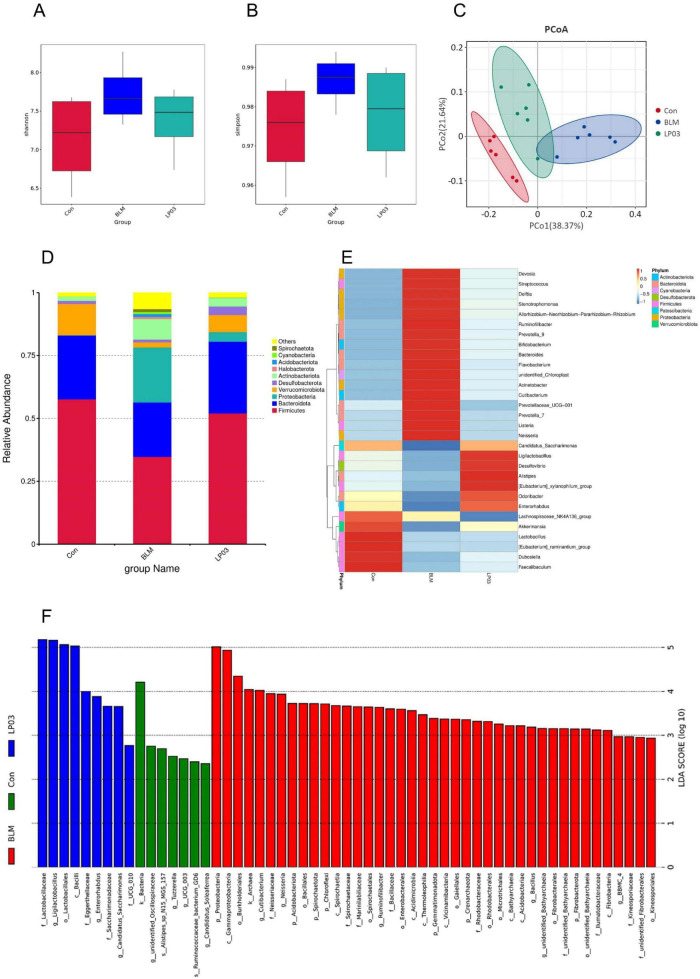
*Lactiplantibacillus* sp. LP03 (LP03) treatment ameliorates gut microbiota dysbiosis in a mouse model of bleomycin (BLM)-induced pulmonary fibrosis. **(A,B)** α-diversity of the gut microbiota across the three groups was assessed using the Shannon and Simpson indices. **(C)** β-diversity was evaluated using Principal Coordinates Analysis (PCoA) based on weighted UniFrac distances among the groups. **(D)** Relative abundance of gut microbiota at the phylum level in the three experimental groups. **(E)** Heatmap showing clustering of gut microbiota relative abundance at the genus level across groups. **(F)** Bar graph displaying linear discriminant analysis (LDA) scores for taxa with significantly different abundances among groups. Only taxa with LDA scores (log10) > 2 and *p* < 0.05 are shown.

To elucidate the underlying mechanisms by which LP03 mitigates BLM-induced pulmonary fibrosis, we investigated the gut microbiota composition across three experimental groups through 16S rDNA sequencing. Microbial α-diversity, evaluated using the Shannon and Simpson indices to assess community richness and evenness, showed no statistically significant differences across study groups (*p* > 0.05). Nevertheless, LP03 administration was found to partially restore the gut microbiota diversity to levels comparable to the Con group in BLM-treated mice (Figures 4A, B). Additionally, β-diversity was assessed using Principal Coordinates Analysis (PCoA) based on weighted UniFrac distances, which revealed a distinct separation between the Con and BLM groups. The microbiota of the LP03 group, however, exhibited an intermediate profile, indicating that LP03 mitigates the dysbiosis induced by BLM (Figure 4C). To explore specific alterations in microbial community structure among the groups, we analyzed and presented the relative abundance of the top ten phyla (Figure 4D). The analysis revealed that BLM treatment caused an imbalance in the phylum-level composition of the gut microbiota, most notably a decrease in the proportion of *Firmicutes* and an increase in *Proteobacteria*. However, LP03 treatment largely restored the structural composition of the gut microbiota. At the genus level, a heatmap depicting the relative abundance of the top thirty genera across the groups was generated (Figure 4E). This analysis revealed that BLM treatment reduced the abundance of beneficial genera such as *Ligilactobacillus* and *Akkermansia*, while increasing the abundance of harmful genera like *Listeria* and *Acinetobacter*. However, LP03 treatment effectively reversed these trends. Furthermore, LEfSe analysis identified distinct gut microbiota species with significantly increased abundance in each group, as shown in Figure 4F.

### 3.5 LP03 gavage altered the metabolic profile of serum samples and increased PEA levels in mice following BLM administration

**FIGURE 5:**
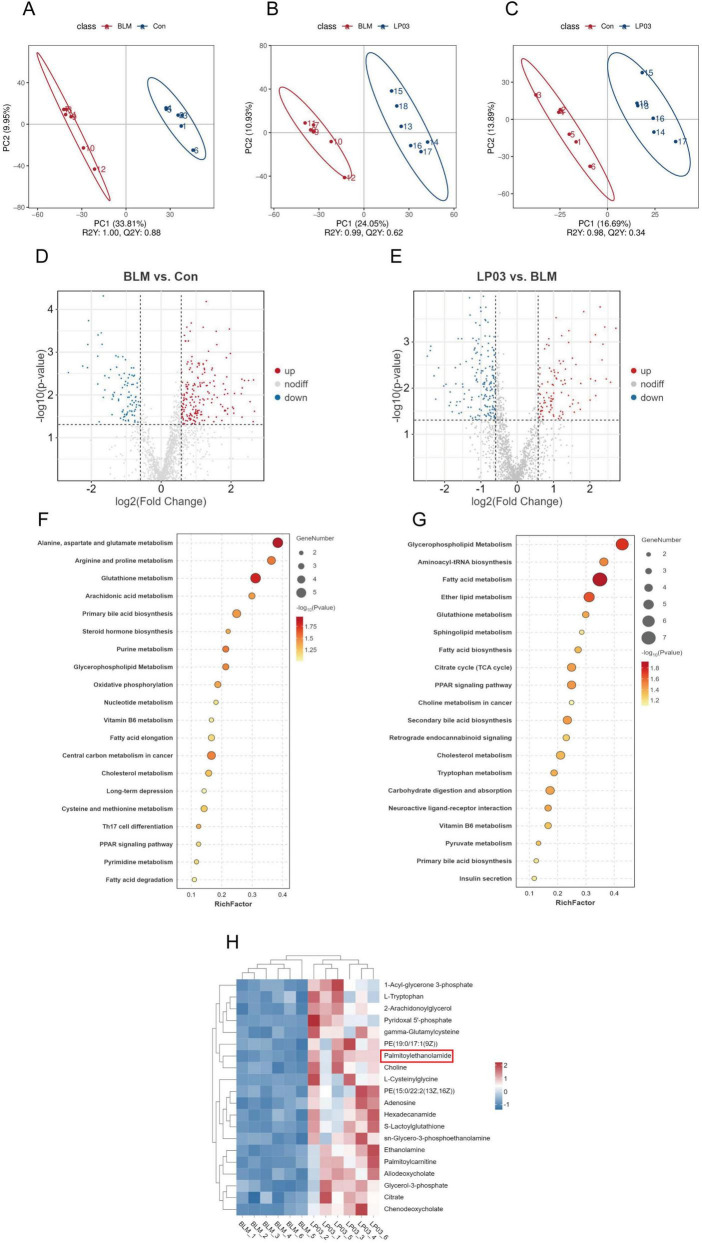
Non-targeted metabolomics profiling of serum. **(A–C)** Partial Least Squares Discriminant Analysis (PLS-DA) of differential metabolites in the serum from the control (Con) and bleomycin (BLM) groups, the BLM and *Lactiplantibacillus* sp. LP03 (LP03) groups, and the Con and LP03 groups. **(D)** Volcano plot illustrating the differential metabolites identified between the Con and BLM groups. **(E)** Volcano plot depicting the differential metabolites identified between the BLM and LP03 groups. **(F)** KEGG pathway enrichment analysis for the Con and BLM groups. **(G)** KEGG pathway enrichment analysis for the BLM and LP03 groups. **(H)** Hierarchical clustering of the top 20 upregulated metabolites in the LP03 versus BLM groups. *p < 0.05, **p < 0.01, ***p < 0.001.

To investigate the mechanism by which LP03 alleviates pulmonary fibrosis in mice, we employed LC-MS to analyze the serum of the mice. Partial Least Squares Discriminant Analysis (PLS-DA) revealed significant differences in metabolite levels among the three experimental groups, incorporating data from both positive and negative ion modes (Figures 5A–C). To further elucidate the effects of LP03 on the modulation of serum metabolites in BLM-treated mice, we performed comparative analyses between the BLM and Con groups, as well as between the LP03 and BLM groups. The comparative analyses were based on thresholds for fold change (FC) > 1.5 and *p*-values < 0.05. As depicted in the volcano plot, 187 metabolites were found to be upregulated and 93 metabolites downregulated in the BLM group relative to the Con group (Figure 5D). Moreover, in the LP03 group compared to the BLM group, 100 metabolites were increased, while 170 metabolites were decreased (Figure 5E).

Kyoto Encyclopedia of Genes and Genomes (KEGG) pathway enrichment analysis demonstrated that BLM administration induced significant alterations in metabolic pathways, particularly disrupting amino acid and lipid metabolism (Figure 5F). Additionally, LP03 treatment mainly modified lipid metabolism, with marked alterations in glycerophospholipid and fatty acid metabolic pathways (Figure 5G). We hypothesized that the therapeutic effects of LP03 in BLM-induced pulmonary fibrosis are mediated through serum metabolite alterations. To identify metabolites with potential antifibrotic activity, we performed hierarchical clustering analysis of the top 20 upregulated metabolites in LP03-treated mice compared to BLM controls (Figure 5H). Among these candidates, palmitoylethanolamide (PEA) emerged as a promising mediator, as this lipid metabolite has been previously established to exert antifibrotic effects in pulmonary, retinal, and hepatic fibrosis (Di Paola et al., 2016; Ohara et al., 2018; Ye et al., 2020). These results suggest that LP03 modulates host lipid metabolism and elevates systemic levels of PEA in the BLM-induced pulmonary fibrosis mouse model. Based on these findings, we propose that PEA mediates, at least in part, the attenuation of BLM-induced pulmonary fibrosis. Subsequent investigations into LP03’s antifibrotic mechanisms have consequently focused on PEA’s therapeutic role.

### 3.6 Administration of PEA ameliorated pulmonary fibrosis in the BLM-induced mouse model by inhibiting EMT and TGF-β1/Smad2/3 pathway

**FIGURE 6:**
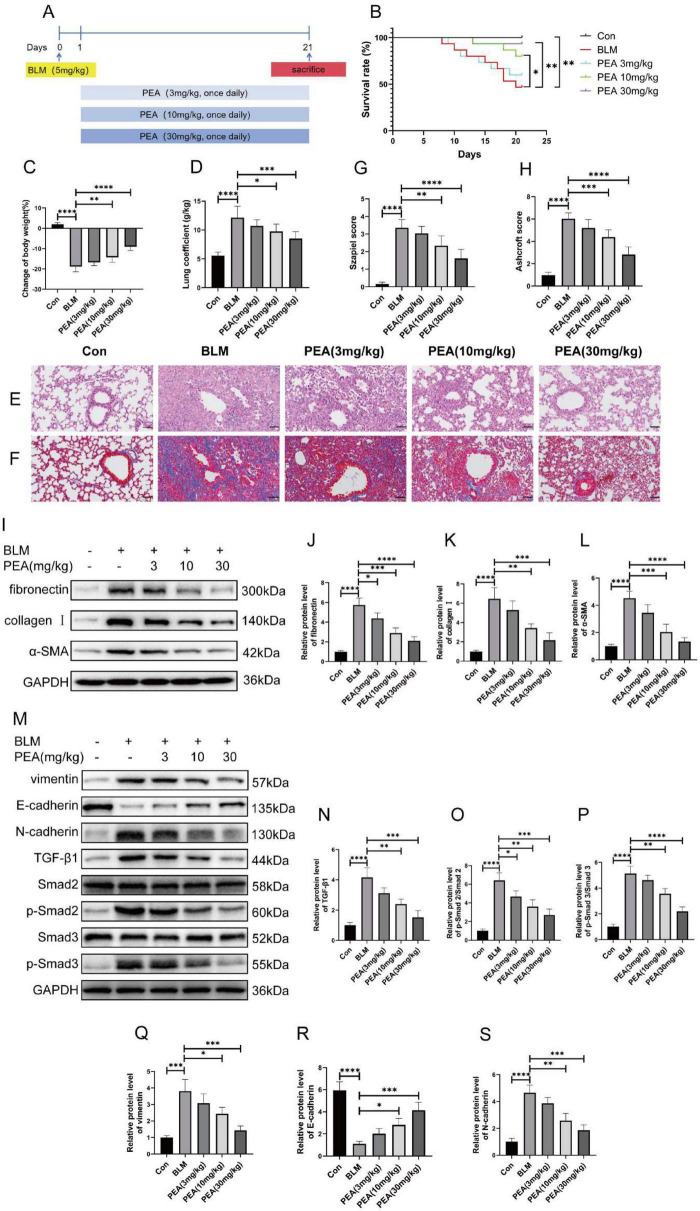
Palmitoylethanolamide (PEA) ameliorates bleomycin (BLM)-induced pulmonary fibrosis in mice and inhibits epithelial-to-mesenchymal transition (EMT) and the TGF-β1/Smad2/3 signaling pathway in a dose-dependent manner. **(A)** Schematic representation of the experimental design illustrating the effect of PEA on BLM-induced pulmonary fibrosis in mice. **(B)** Kaplan-Meier survival curves showing the survival rates of mice in each group throughout the experimental period. **(C)** Endpoint body weight changes across the different groups. **(D)** Lung coefficient of mice at the time of sacrifice. **(E,F)** Representative histopathological images of lung tissues from mice at the study endpoint, stained with hematoxylin and eosin (H&E) and Masson’s trichrome (Scale bar = 50 μm). **(G,H)** Quantitative assessment of lung tissue histology based on Szapiel and Ashcroft scores for H&E and Masson’s trichrome-stained sections, respectively. **(I–L)** Relative protein expression levels of fibrotic markers assessed by western blot and quantified using ImageJ software. **(M–S)** Relative protein expression levels of EMT-related markers, TGF-β1, and phosphorylated Smad2/3, determined by western blot and analyzed using ImageJ software. Data are presented as mean ± SD; *p < 0.05, **p < 0.01, ***p < 0.001, ****p < 0.0001 vs. BLM group.

Next, we investigated whether PEA mitigates BLM-induced pulmonary fibrosis. Figure 6A is a schematic diagram of the experimental design. We found that oral administration of PEA effectively improved the survival rate of mice in the pulmonary fibrosis model (Figure 6B), mitigated weight loss induced by BLM (Figure 6C), and reduced the lung coefficient (Figure 6D), exhibiting a clear dose-dependent effect. Moreover, histopathological analysis of lung tissue, using H&E and Masson’s trichrome staining, confirmed that PEA markedly alleviated pulmonary inflammatory cell infiltration and collagen fiber deposition in the mice (Figures 6E–H). Additionally, western blot analysis revealed that PEA significantly reduced the expression levels of fibronectin, collagen I, and α-SMA in a dose-dependent manner in the pulmonary fibrosis mouse model (Figures 6I–L). To further investigate whether PEA alleviates pulmonary fibrosis in mice by modulating the TGF-β1/Smad2/3 pathway and EMT, we performed western blot analysis to assess the expression levels of EMT-related markers, TGF-β1, and the phosphorylation status of Smad2/3. The results revealed that PEA significantly reduced the ratios of p-Smad2/Smad2 and p-Smad3/Smad3, as well as the expression level of TGF-β1, in a dose-dependent manner (Figures 6M–P). These findings indicate that PEA effectively suppresses the TGF-β1/Smad2/3 signaling pathway activated in BLM-induced pulmonary fibrosis. Furthermore, PEA significantly reversed the elevated expression levels of vimentin and N-cadherin, and the decreased expression of E-cadherin observed in the BLM group, suggesting that PEA efficiently inhibits BLM-induced EMT *in vivo* (Figures 6M, Q–S).

### 3.7 PEA attenuates TGF-β1-induced fibrosis-associated gene expression and EMT by inhibiting Smad2/3 phosphorylation *in vitro*

**FIGURE 7:**
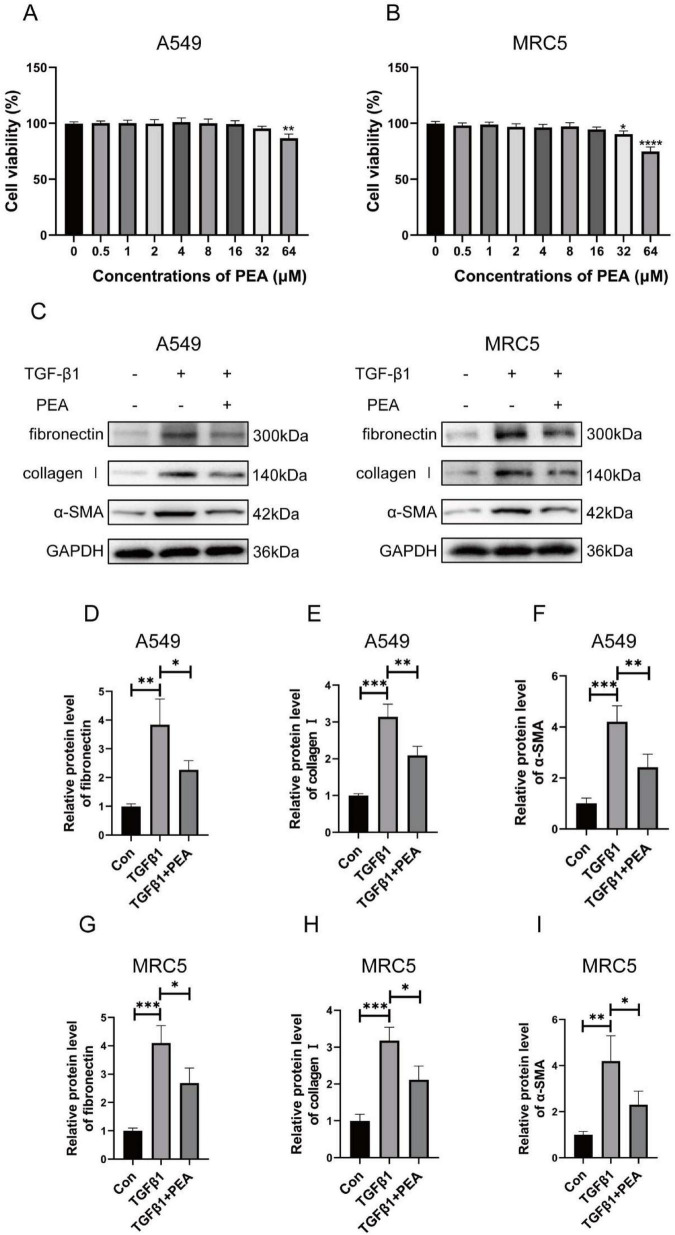
Palmitoylethanolamide (PEA) reduced the expression of fibrosis-related genes in A549 and MRC5 cell lines stimulated by TGF-β1. **(A,B)** Cell viability of A549 and MRC5 cell lines following PEA treatment was assessed using the MTT assay. **(C–I)** The relative protein expression levels of fibronectin, collagen I, and α-SMA in both cell lines were quantified by western blotting and analyzed using ImageJ software. Data are presented as mean ± SD; *p < 0.05, **p < 0.01, ***p < 0.001, ****p < 0.0001.

Subsequently, we investigated whether PEA attenuates fibrosis-associated gene expression and EMT in an *in vitro* model of pulmonary fibrosis, utilizing the human bronchial epithelial cell line A549 and the human lung fibroblast cell line MRC5. To assess the *in vitro* toxicity of PEA, A549 and MRC5 cell lines were exposed to a range of PEA concentrations. As shown in Figures 7A, B, PEA demonstrated no significant cytotoxicity to the A549 cell line at concentrations up to 32 μM, and no significant cytotoxicity to the MRC5 cell line at concentrations up to 16 μM. Based on the concentrations of TGF-β1 used in cell models of pulmonary fibrosis and PEA in *in vitro* experiments as reported in the literature, we selected a concentration of 5 ng/mL for TGF-β1 to stimulate the cells and 10 μM for PEA in the subsequent experiments (Ohara et al., 2018; Wang et al., 2020; Schiano Moriello et al., 2022; Niu et al., 2023; Ma et al., 2024). We then investigated the effect of PEA on the expression of fibrosis-related genes in the A549 and MRC5 cell lines under TGF-β1 stimulation. Our results demonstrated that PEA significantly reduced the expression levels of fibronectin, collagen I, and α-SMA in A549 (Figures 7C–F) and MRC5 (Figures 7C, G–I) cell lines, consistent with previous *in vivo* findings, thereby further confirming the potential antifibrotic effects of PEA.

**FIGURE 8:**
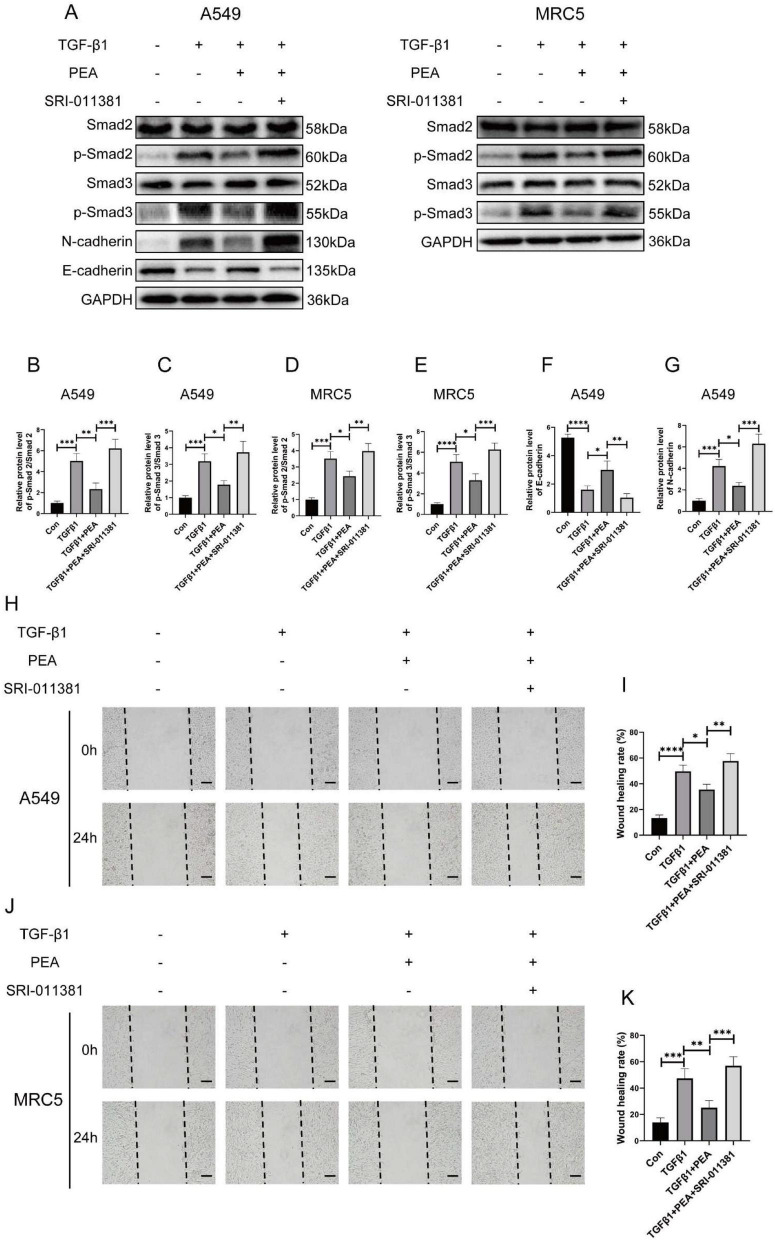
Palmitoylethanolamide (PEA) inhibits TGF-β1-induced cellular transition to the mesenchymal phenotype by suppressing Smad2/3 phosphorylation *in vitro*. **(A–G)** The relative protein expression levels of p-Smad2/3 in A549 and MRC5 cells, as well as E-cadherin and N-cadherin in A549 cells, were assessed by western blotting and analyzed using ImageJ software. **(H–K)** The effect of PEA on the migration of A549 and MRC5 cells stimulated by TGF-β1 was assessed using a wound healing assay, with quantitative analysis performed via ImageJ software (scale bar = 200 μm). Data are presented as mean ± SD; *p < 0.05, **p < 0.01, ***p < 0.001, ****p < 0.0001.

To investigate whether PEA modulates the expression of fibrosis-related genes by inhibiting TGF-β1/Smad2/3 signaling-mediated EMT, we supplemented the TGF-β/Smad signaling pathway agonist SRI-011381 in TGF-β1- and PEA-treated cell lines. Western blot analysis demonstrated that PEA attenuated the phosphorylation of Smad2 and Smad3 induced by TGF-β1 in both A549 and MRC5 cell lines, while SRI-011381 reversed this effect of PEA (Figures 8A–E). Furthermore, in the A549 cell line, PEA reduced the expression of N-cadherin induced by TGF-β1 and increased the expression of E-cadherin, which was suppressed by TGF-β1. Notably, SRI-011381 reversed the effect of PEA on the expression of these EMT-related markers (Figures 8A, F, G). Additionally, PEA significantly reduced the mobility of TGF-β1-stimulated A549 and MRC5 cells, as shown by the wound healing assay, which was reversed by SRI-011381 (Figures 8H–K). These findings support the conclusion that PEA inhibits the acquisition of a mesenchymal phenotype in TGF-β1-stimulated alveolar epithelial cells and pulmonary fibroblasts by suppressing Smad2/3 phosphorylation. In summary, PEA inhibits TGF-β1-induced phosphorylation of Smad2/3, thereby suppressing EMT and the expression of fibrosis-related genes *in vitro*.

## 4 Discussion

**FIGURE 9:**
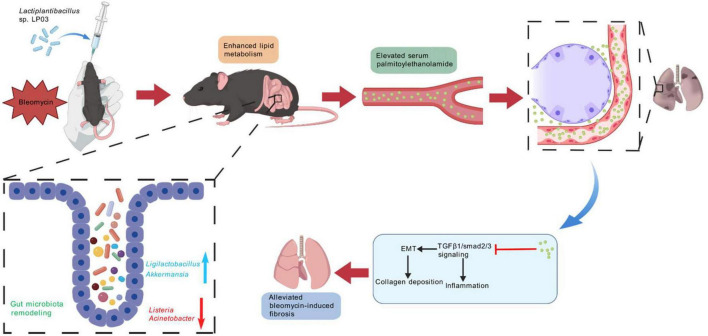
*Lactiplantibacillus* sp. LP03 (LP03) attenuates bleomycin (BLM)-induced pulmonary fibrosis by modulating gut microbiota composition and enhancing palmitoylethanolamide (PEA0 levels, which inhibit epithelial-to-mesenchymal transition (EMT) via suppression of the TGF-β1/Smad2/3 signaling pathway.

Pulmonary fibrosis is a chronic, progressive interstitial lung disease characterized by high mortality. The management of this condition remains a significant clinical challenge, owing to the complexity of its underlying pathogenic mechanisms. In this study, we identified LP03, a probiotic strain isolated from traditional fermented sauerkraut juice, as a promising therapeutic candidate for attenuating BLM-induced pulmonary fibrosis in a murine model. The observed therapeutic effects appear to be mediated by the enrichment of beneficial gut microbiota (e.g., *Ligilactobacillus* and *Akkermansia*), suppression of potentially pathogenic bacteria (e.g., *Listeria* and *Acinetobacter*), and, most notably, the regulation of PEA, a protective lipid metabolite. As demonstrated in our study, exogenous administration of PEA provided protection against BLM-induced pulmonary fibrosis, with efficacy comparable to LP03 intervention in the murine model. Furthermore, *in vitro* and *in vivo* experiments confirmed that PEA alleviated BLM-induced pulmonary fibrosis by inhibiting the TGF-β1/Smad2/3 signaling pathway and suppressing EMT (Figure 9).

The gut-lung axis refers to the bidirectional communication between the gastrointestinal system and the lungs, with the gut microbiota playing a crucial role in modulating both lung health and disease (Alswat, 2024). Research has shown that disruptions in gut microbiota are closely linked to the onset and progression of various respiratory diseases, including asthma, COPD, pulmonary fibrosis, and acute lung injury (Chen Z. et al., 2024; Ruan et al., 2024; Song et al., 2024; Zhang J. et al., 2024). Modulating and restoring the gut microbiota has thus become a therapeutic target for various pharmaceuticals and probiotic formulations aimed at treating respiratory conditions. For example, a randomized controlled pilot study demonstrated that the probiotic *Pro-Vi 5* improved quality of life and modulated immune responses in patients with severe COVID-19, potentially through the gut-lung axis (Horvath et al., 2024). In preclinical studies, *Lactobacillus rhamnosus* modulated lung inflammation and alleviated gut dysbiosis in a murine model of asthma-COPD overlap syndrome (Vasconcelos et al., 2023). Similarly, *Lactobacillus reuteri* alleviated lipopolysaccharide-induced acute lung injury in mice through gut microbiota modulation (Shen et al., 2023). Notably, the therapeutic effects of 20(S)-protopanaxadiol, umbilical cord-derived mesenchymal stem cells, kefir peptides and the traditional Chinese medicine Bu-Fei-Huo-Xue capsule in mitigating BLM-induced pulmonary fibrosis in mice are all closely tied to their modulation of gut microbiota (Hu et al., 2023; Lan et al., 2024; Ruan et al., 2024; Luo et al., 2025). Unlike *Lactobacillus rhamnosus GG*, which was shown to attenuate radiation-induced pulmonary fibrosis through direct regulation of the lncRNA SNHG17/PTBP1/NICD axis, LP03 exerts its antifibrotic effects by partially restoring the disrupted gut microbiota composition in the pulmonary fibrosis model—enriching beneficial genera (e.g., *Ligilactobacillus*, *Akkermansia*) while reducing pathogenic genera (e.g., *Listeria*, *Acinetobacter*)—and by modulating host lipid metabolism, particularly through elevating endogenous PEA levels and suppressing TGF-β1/Smad2/3 signaling. This unique mechanism highlights a novel link between microbial ecology and host metabolic signaling, distinguishing LP03 from other *Lactobacillus* strains investigated in pulmonary fibrosis research. Moreover, while conventional antifibrotic agents such as pirfenidone mitigate EMT and extracellular matrix deposition primarily by directly modulating pulmonary Wnt/GSK-3β/β-catenin and TGF-β1/Smad2/3 signaling pathways, LP03 offers complementary benefits by linking gut microbiota modulation to systemic metabolic regulation (Lv et al., 2020).

Furthermore, our study demonstrates that LP03 administration significantly enhances lipid metabolism in a BLM-induced pulmonary fibrosis mouse model, particularly affecting the glycerophospholipid and fatty acid metabolic pathways. LP03 treatment markedly increased serum levels of various lipid metabolites, including PEA. Elevated PEA levels were accompanied by increased abundance of certain beneficial genera such as *Ligilactobacillus* and *Akkermansia*, raising the possibility of gut microbiota-mediated regulation. Notably, a previous study suggested that alterations in the gut microbiota may contribute to disease development by modulating PEA levels. A population-based cohort study revealed that PEA acts as a mediator linking gut microbial diversity to anhedonia and amotivation (Minichino et al., 2021). Moreover, in a mouse model of hepatic steatosis induced by a high-methionine diet, levels of bioactive lipids, including PEA, were found to correlate with the abundance of several gut bacterial genera such as *Solitalea*, further supporting the potential involvement of gut microbiota in regulating lipid metabolism and PEA levels (Zhou L. et al., 2024). However, the mechanistic link between gut microbial composition and endogenous PEA levels remains largely unclear and requires further investigation.

Palmitoylethanolamide, an endogenous *N*-acylethanolamine, exhibits pleiotropic biological effects, including potent anti-inflammatory, analgesic, immunomodulatory, and neuroprotective properties (Clayton et al., 2021; Argueta et al., 2025). The synthesis of PEA is a lipid-driven, enzyme-regulated process that connects phospholipids to anti-inflammatory signaling pathways (Natarajan et al., 1983; Schmid et al., 1983). Preliminary evidence suggests that PEA possesses antifibrotic effects in pulmonary, hepatic, and retinal fibrosis, supporting its potential role in mediating LP03’s therapeutic effects (Di Paola et al., 2016; Ohara et al., 2018; Ye et al., 2020). However, the existing research on the antifibrotic properties of PEA remains limited, necessitating further investigation. Given PEA’s high lipophilicity and poor water solubility, enhancing its bioavailability following exogenous administration via the gastrointestinal tract typically requires micronization (Petrosino et al., 2018; Annunziata et al., 2020). This process increases the surface area of PEA particles, thereby improving their absorption into the bloodstream. In this study, we employed ultra-micronized PEA for oral gavage in mice to enhance its bioavailability.

Accumulating evidence suggests that certain *Lactobacillus* species can modulate host metabolism, contributing to their therapeutic potential in various diseases. For instance, *Lactobacillus* has been shown to alleviate metabolic dysfunction-associated steatotic liver disease by modulating the gut microbiota, regulating hepatic lipid metabolism, and improving insulin resistance (Olotu and Ferrell, 2024). *Lactobacillus plantarum* mitigates glucocorticoid-induced osteoporosis by modulating the composition of the rat gut microbiota and altering the serum metabolic profile (Li S. et al., 2023). The probiotics *Bifidobacterium lactis* M8 and *Lactobacillus rhamnosus* M9 prevent hypertension through modulation of the gut microbiota composition and the regulation of host metabolic products (Zhang Y. et al., 2023). Additionally, *Lactobacillus plantarum* AR495 alleviates colonic transport hyperactivity in irritable bowel syndrome by modulating tryptophan metabolism in colonic tissue (Zhang H. et al., 2024).

TGF-β1 is a multifunctional cytokine that plays a pivotal role in various biological processes, including cell proliferation, differentiation, and extracellular matrix formation (Hu et al., 2018). Its expression is upregulated in the lung tissue of animal models of BLM-induced pulmonary fibrosis (Ding et al., 2023; Zhang R. et al., 2023). During fibrosis, TGF-β1 initiates intracellular signaling by binding to cell membrane receptors, which leads to the phosphorylation of Smad2 and Smad3. These phosphorylated Smads then form heterotrimeric complexes with Smad4, which translocate to the nucleus and bind to specific DNA sequences, thereby regulating the expression of EMT-related genes. Consequently, the TGF-β/Smad signaling pathway plays a critical role in the pathogenesis of pulmonary fibrosis and serves as a key therapeutic target for antifibrotic drugs (Du et al., 2024; Tirunavalli and Andugulapati, 2024; Wang et al., 2024). The antifibrotic effects of PEA in hepatic and retinal fibrosis have been attributed to its inhibition of the TGF-β/Smad signaling pathway. PEA alleviates profibrotic changes by suppressing the phosphorylation of Smad2/3 in both TGF-β2-treated rat Müller cells and an oxygen-induced retinopathy mouse model (Ye et al., 2020). Moreover, in a study investigating the alleviation of carbon tetrachloride-induced liver fibrosis in rats, PEA was shown to reduce the phosphorylation of Smad2 in TGF-β1-stimulated LX-2 hepatic stellate cells and significantly inhibit the transcriptional activity of Smad complexes (Ohara et al., 2018). Notably, our study is the first to demonstrate that PEA exerts its antifibrotic effects in pulmonary fibrosis by inhibiting the TGF-β/Smad signaling pathway, thus aligning with previously reported mechanisms through which PEA mitigates other fibrotic diseases.

While this study positions LP03 as a promising therapeutic candidate for pulmonary fibrosis, several limitations warrant consideration. First, although LP03 treatment was associated with concurrent changes in both gut microbiota composition and systemic metabolite profiles (particularly lipid metabolites such as PEA), the causal relationship and underlying mechanisms by which gut microbes influence host lipid metabolism remain unclear. Elucidating these pathways will be essential to support the development of microbiota-targeted therapeutic strategies. Second, while both *in vivo* and *in vitro* experiments demonstrated that PEA attenuates fibrosis by inhibiting the TGFβ1/Smad2/3 signaling pathway, the specific molecular targets and downstream signaling cascades involved require additional exploration. Third, although our findings suggest that LP03 alters the gut microbiota and enhances PEA production, thereby modulating the TGF-β/Smad pathway, the mechanistic linkage among these elements has not been fully established. Future studies employing germ-free or antibiotic-treated mice to isolate the role of the microbiota, applying specific inhibitors or knockout models to validate the involvement of PEA/TGF-β signaling, and conducting detailed metabolomic analyses to identify microbial metabolites directly correlated with PEA production will be essential to delineate this causal network. Fourth, differences between animal models and human physiology may limit translational potential; therefore, validation in human tissues and carefully designed clinical studies will be required. Fifth, although ultra-micronized PEA was used in this study to enhance oral bioavailability, clinical applicability may be constrained by formulation, pharmacokinetics, and dosing considerations, which warrant further pharmacological and clinical optimization. Finally, individual variability in gut microbiota composition may influence the efficacy of LP03, underscoring the importance of personalized or stratified approaches in future translational studies.

## 5 Conclusion

This study identifies LP03 as a novel probiotic strain with therapeutic potential for pulmonary fibrosis. The underlying mechanism involves remodeling of the gut microbiota and subsequent alterations in host metabolism, leading to elevated systemic levels of PEA. By inhibiting the TGF-β1/Smad2/3 signaling pathway and EMT, LP03-mediated enhancement of PEA effectively attenuates fibrotic progression. Importantly, this work provides the first evidence that probiotic intervention can exert antifibrotic effects through PEA regulation of the TGF-β1/Smad2/3 axis, highlighting a unique link between microbial ecology and host metabolic signaling.

Nevertheless, the causal relationship between gut microbiota alterations, PEA elevation, and fibrosis resolution remains to be fully elucidated, and further studies are needed to validate these findings in clinical settings. Future investigations should also explore the mechanistic underpinnings of the gut–PEA–fibrosis axis and assess the long-term efficacy and safety of LP03 as a therapeutic strategy. Collectively, these findings not only expand our understanding of host–microbe–metabolite interactions but also establish a scientific rationale for developing probiotic-based interventions against IPF, a progressive and life-threatening disease.

## Data Availability

The datasets presented in this study can be found in online repositories. The names of the repository/repositories and accession number(s) can be found in the article/Supplementary material.
