# Supplementary material for: Lactiplantibacillus sp. LP03 alleviates pulmonary fibrosis by modulating gut microbiota and elevating host palmitoylethanolamide to suppress TGF-β1/Smad2/3-mediated EMT
Source: Front Microbiol. 2025 Sep 9;16:1659142. doi: 10.3389/fmicb.2025.1659142 (PMC12456189; doi:10.3389/fmicb.2025.1659142)
Supplement: Supplementary file 1 [file Supplementary_file_1.docx]

***Supplementary Figure 1***

Detection of the acid tolerance and bile salt resistance of the three strains.


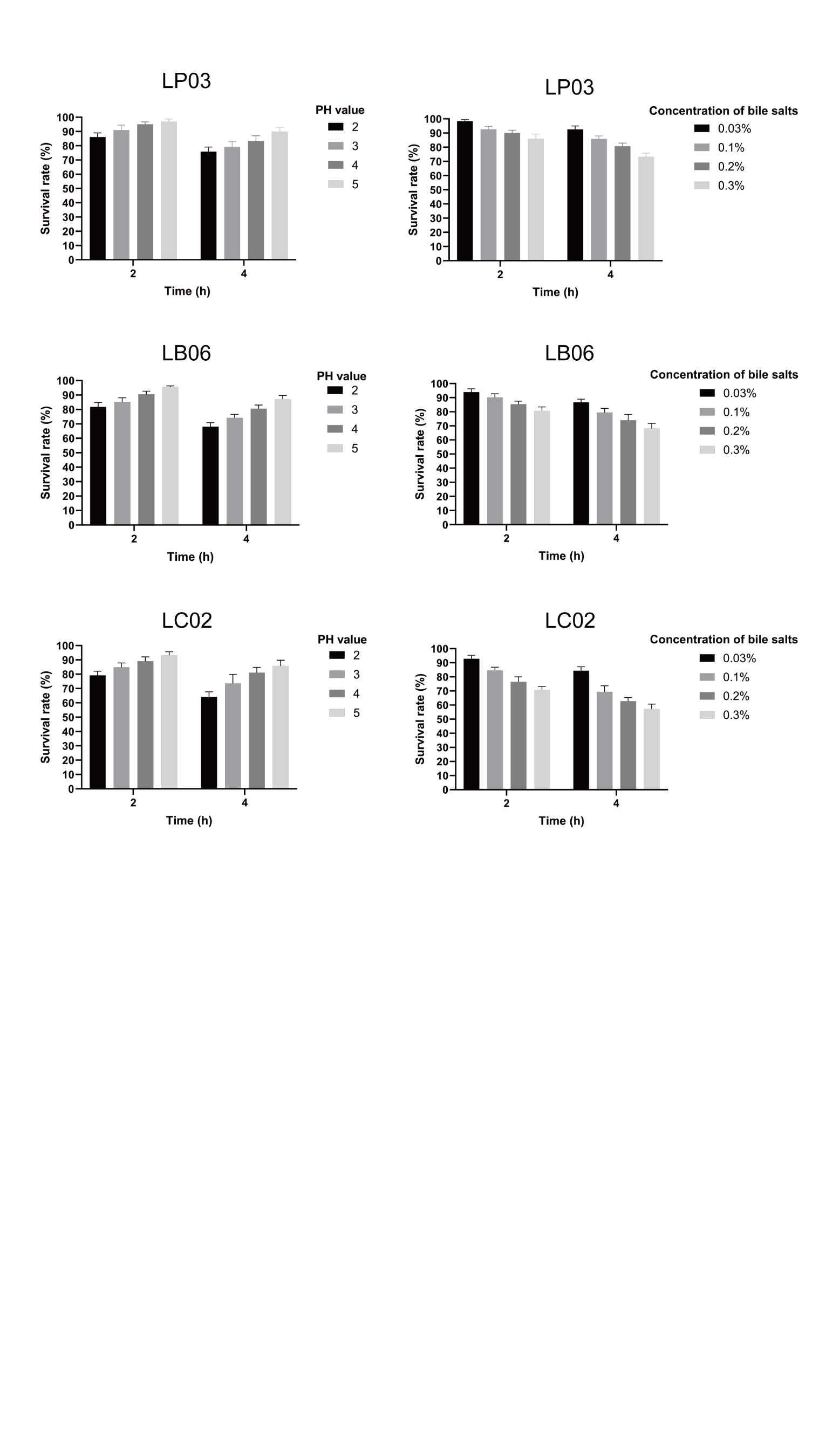


***Supplementary Table 1***

The primer used in RT-qPCR were listed as follows.

| **Gene** | **Forward primer (5′-3′)** | **Reverse primer (5′-3′)** |
| --- | --- | --- |
| *Fibronectin* | GCGACGGTATTCTGTAAAGTGG | GGACAGGGCTTTGGCAGTT |
| *Collagen Ⅰ* | GCTCCTCTTAGGGGCCACT | CCACGTCTCACCATTGGGG |
| *α-SMA* | CCCAACTGGGACCACATGG | TACATGCGGGGGACATTGAAG |
| *Vimentin* | CGTCCACACGCACCTACAG | GGGGGATGAGGAATAGAGGCT |
| *E-cadherin* | CAGGTCTCCTCATGGCTTTGC | CTTCCGAAAAGAAGGCTGTCC |
| *N-cadherin* | AGCGCAGTCTTACCGAAGG | TCGCTGCTTTCATACTGAACTTT |
| *GAPDH* | TGGATTTGGACGCATTGGTC | TTTGCACTGGTACGTGTTGAT |
